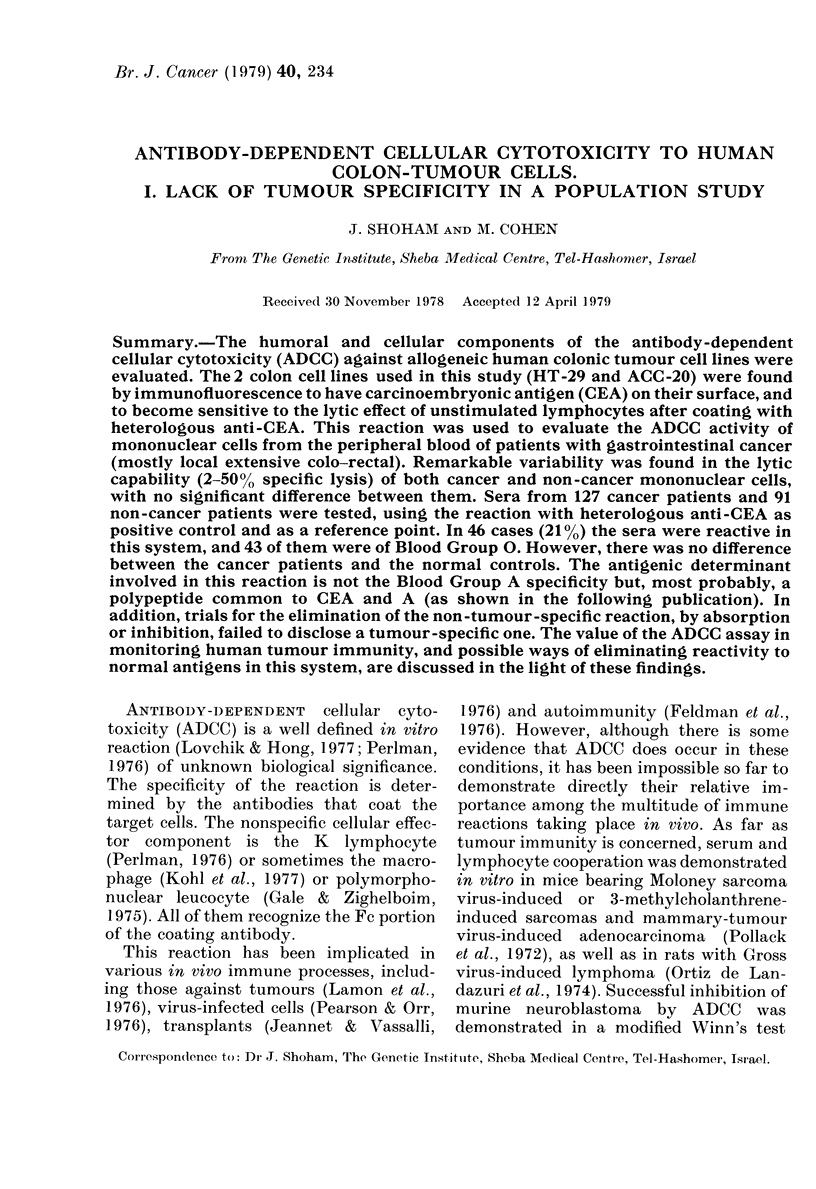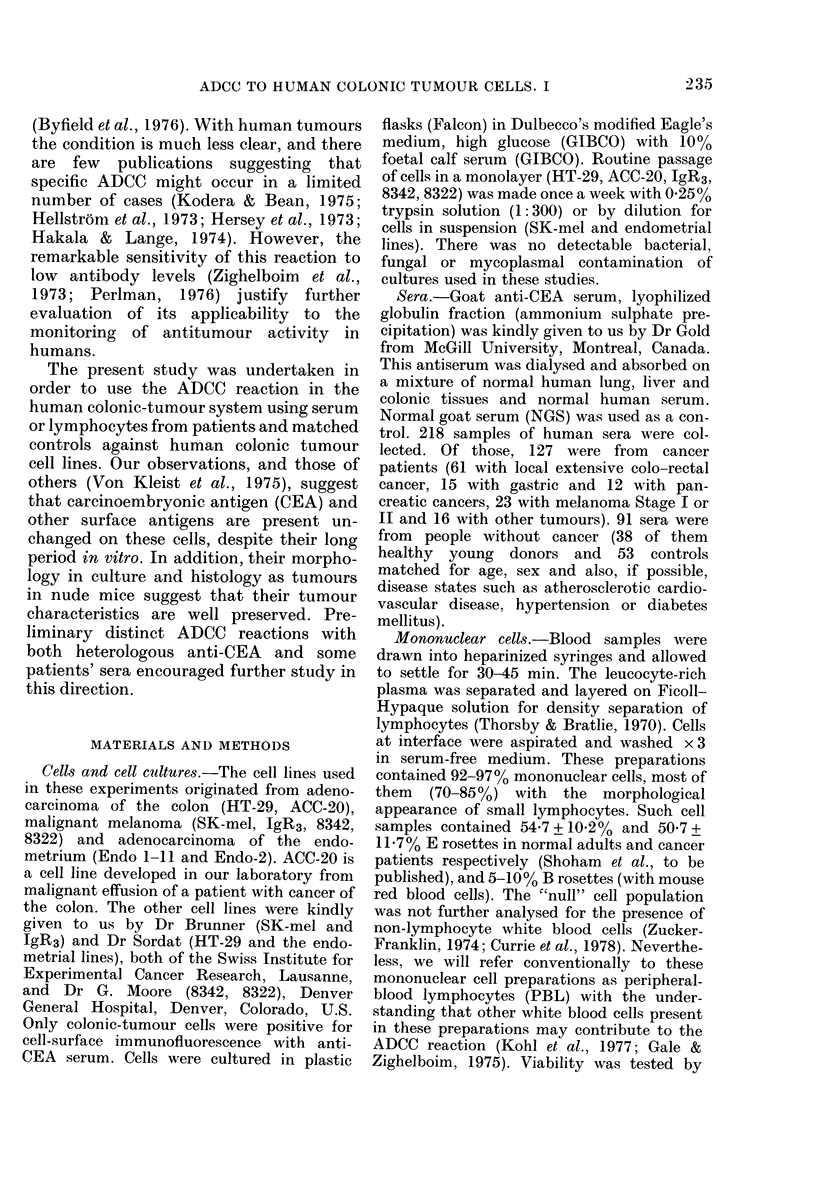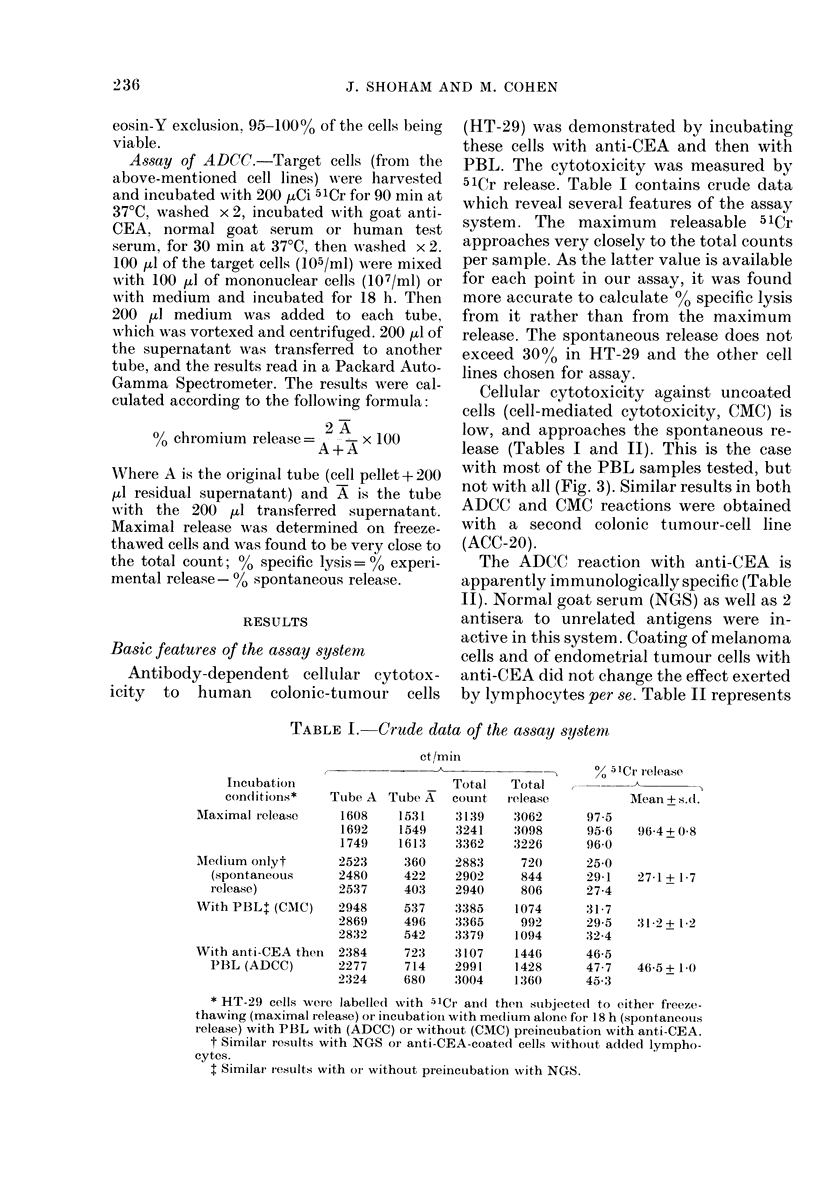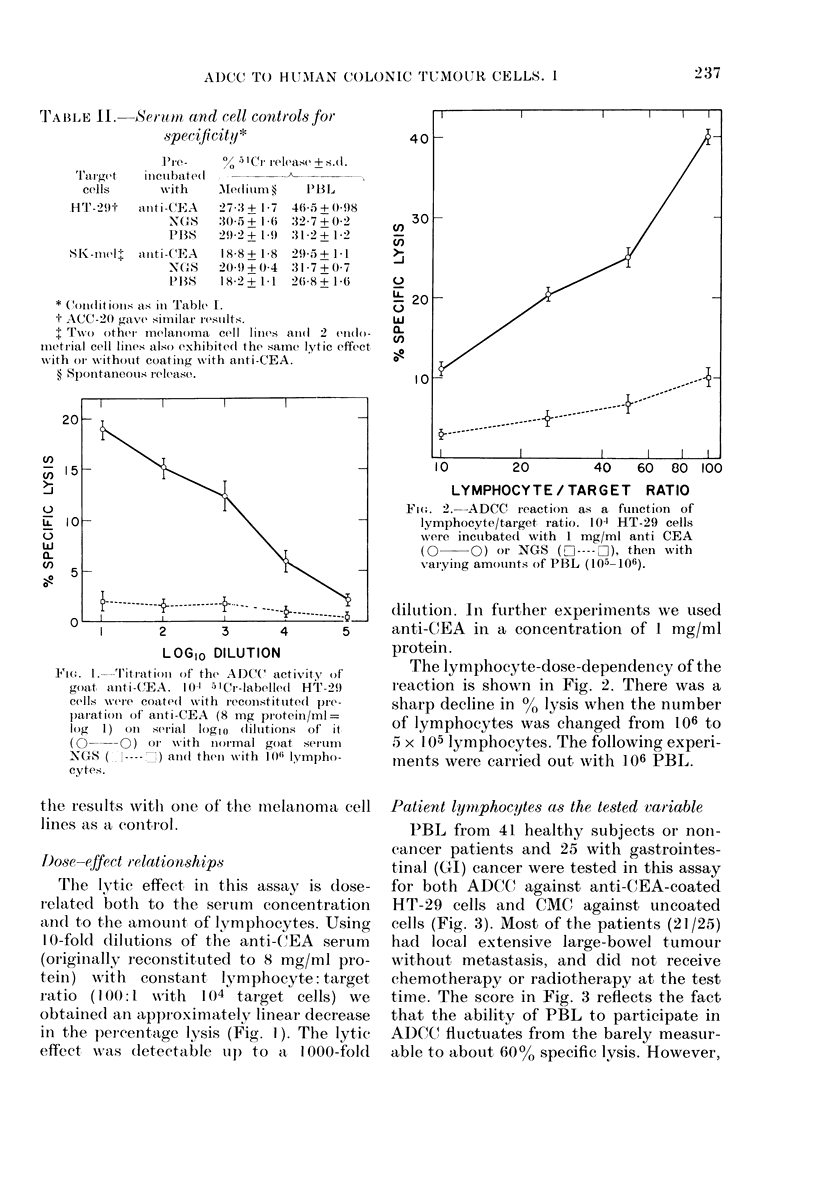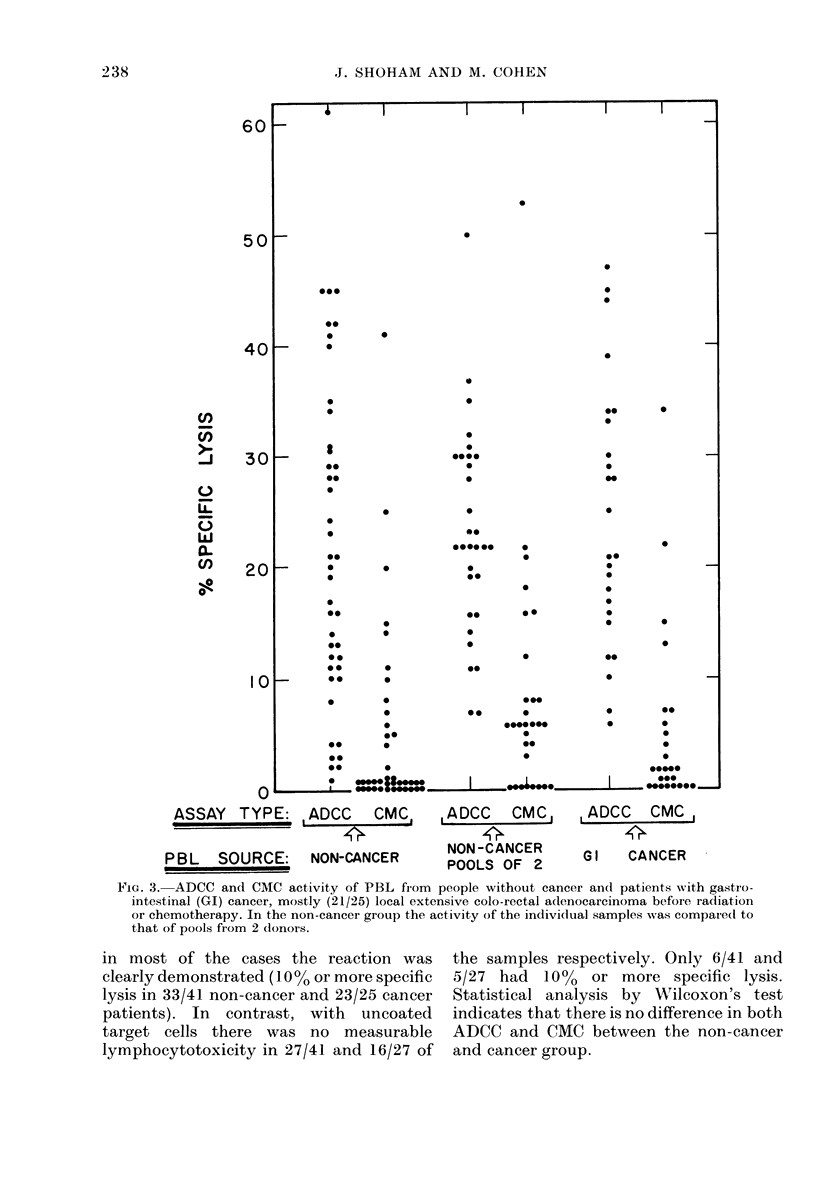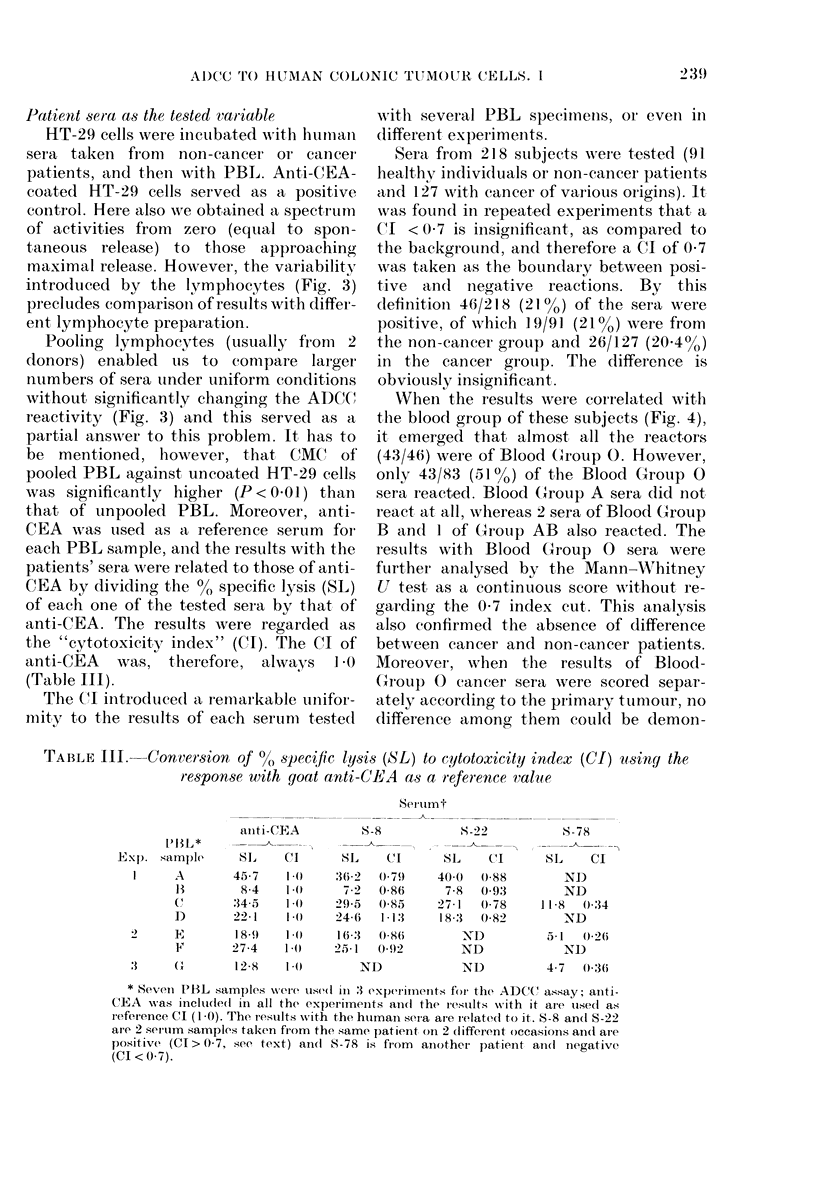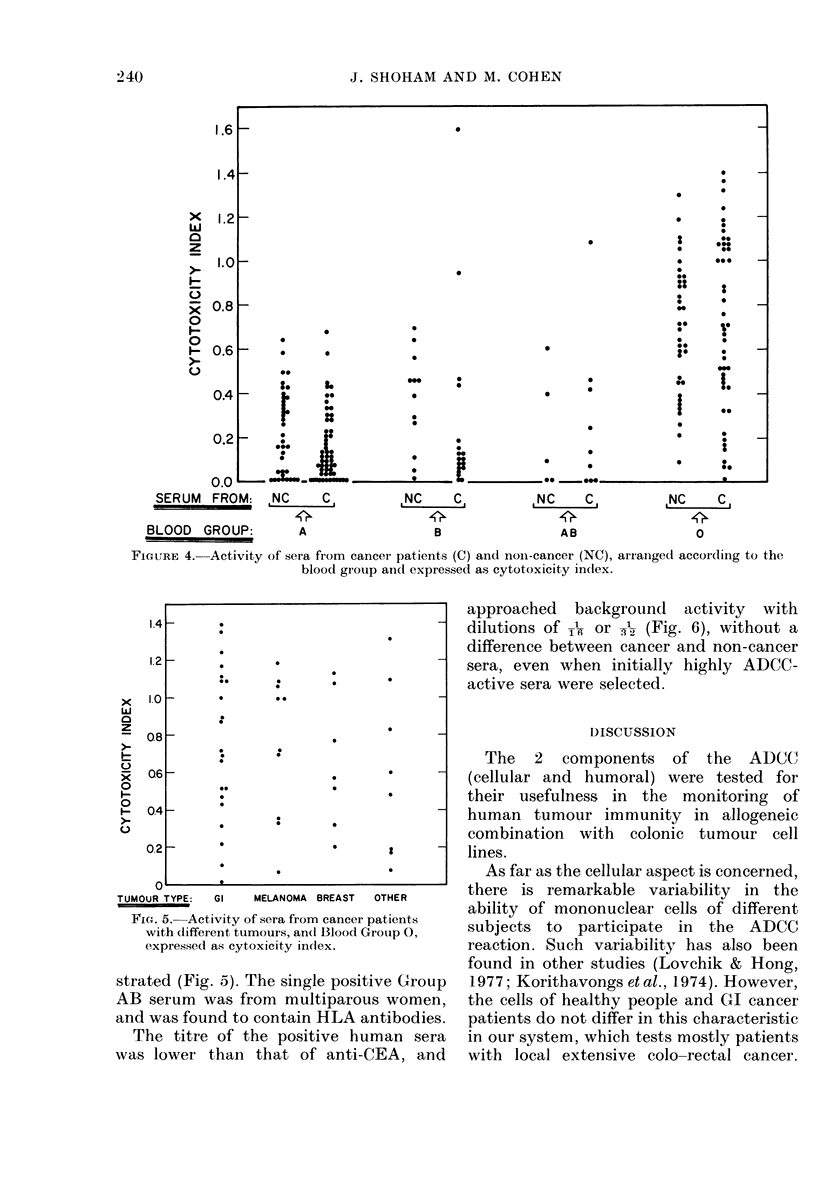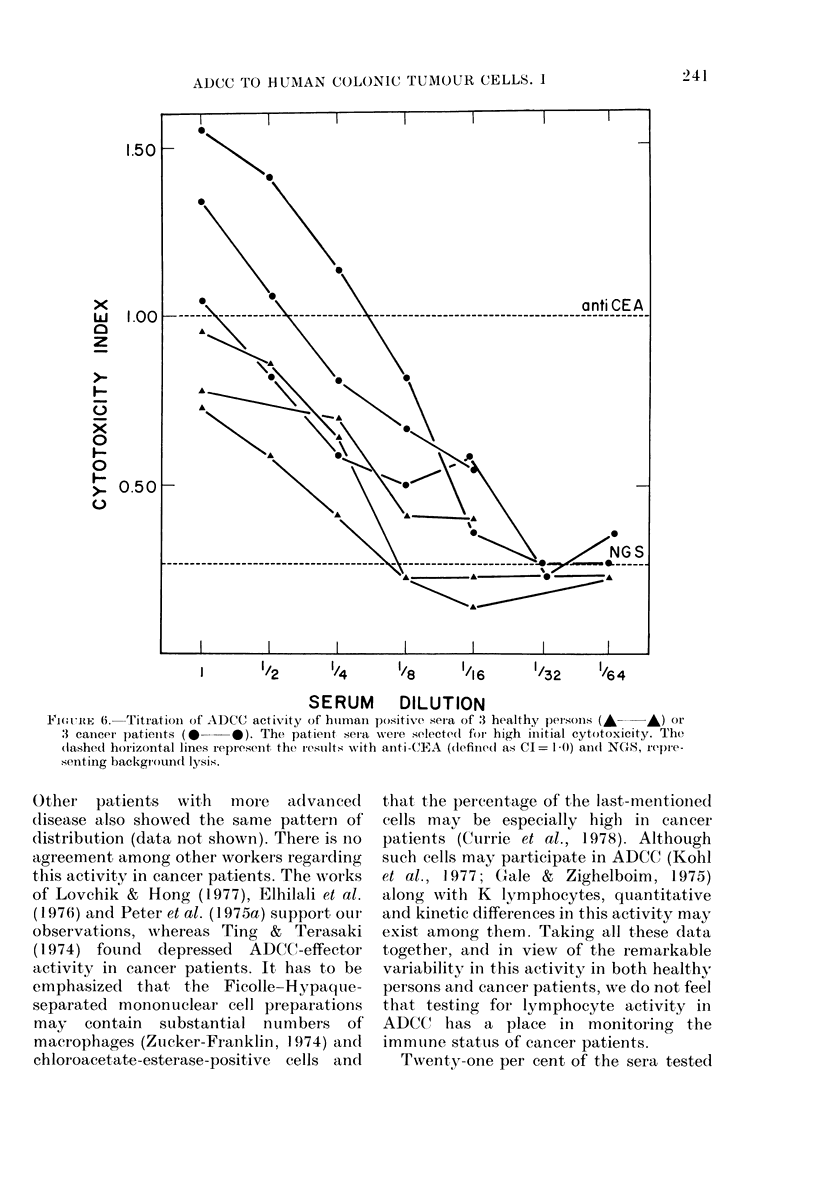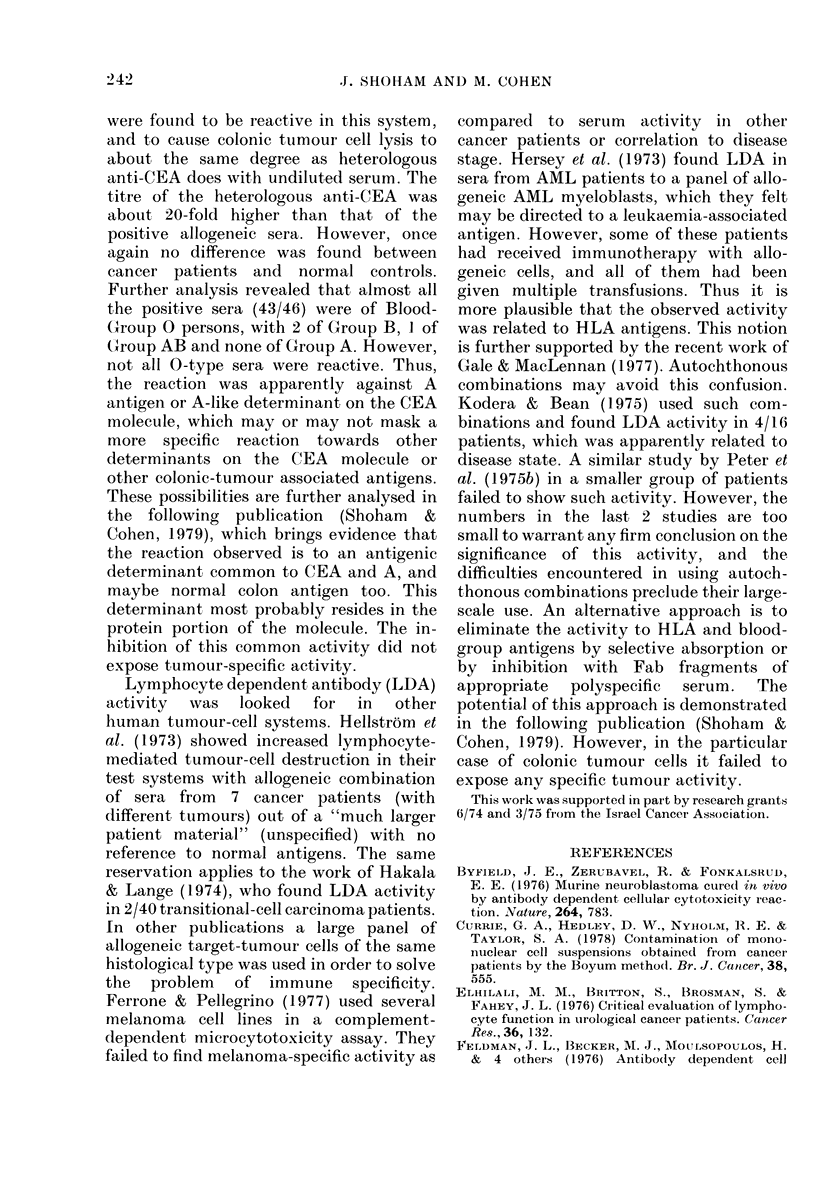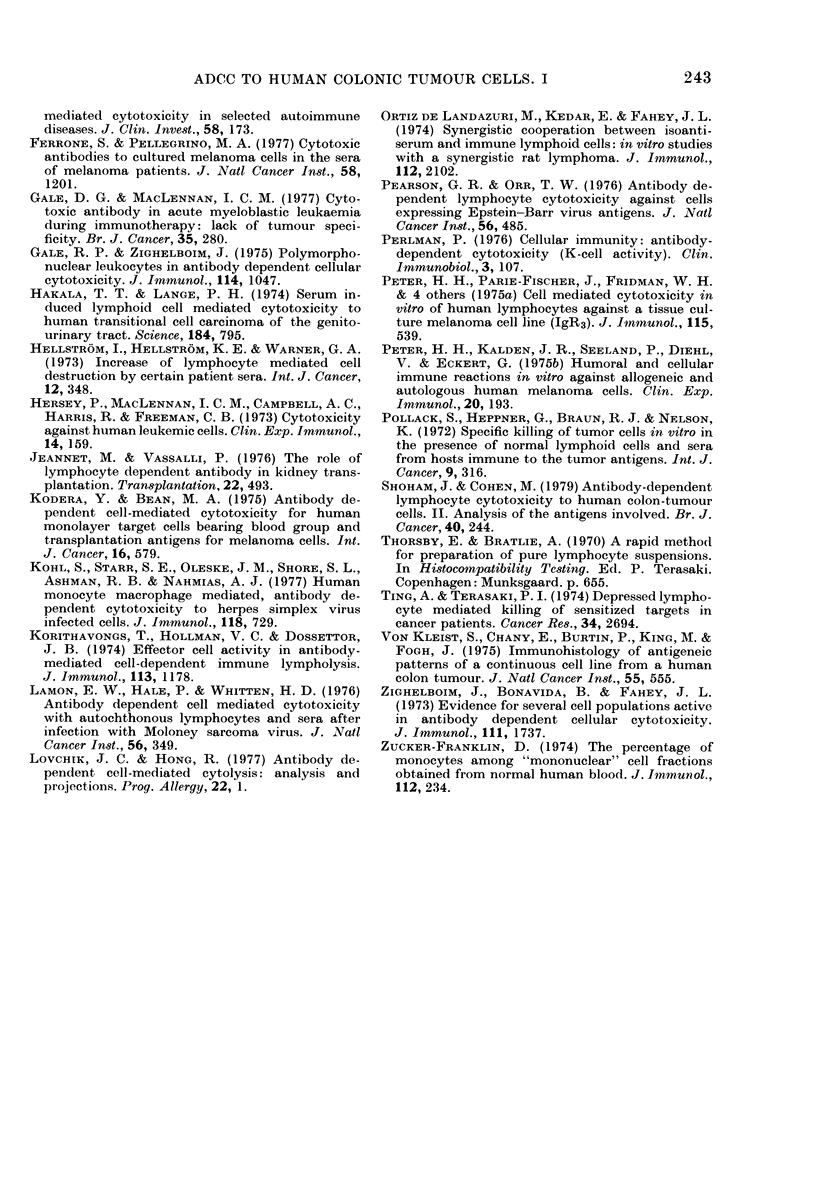# Antibody-dependent cellular cytotoxicity to human colon-tumour cells. I. Lack of tumour specificity in a population study

**DOI:** 10.1038/bjc.1979.171

**Published:** 1979-08

**Authors:** J. Shoham, M. Cohen

## Abstract

The humoral and cellular components of the antibody-dependent cellular cytotoxicity (ADCC) against allogeneic human colonic tumour cell lines were evaluated. The 2 colon cell lines used in this study (HT-29 and ACC-20) were found by immunofluorescence to have carcinoembryonic antigen (CEA) on their surface, and to become sensitive to the lytic effect of unstimulated lymphocytes after coating with heterologous anti-CEA. This reaction was used to evaluate the ADCC activity of mononuclear cells from the peripheral blood of patients with gastrointestinal cancer (mostly local extensive colo-rectal). Remarkable variability was found in the lytic capability (2-50% specific lysis) of both cancer and non-cancer mononuclear cells, with no significant difference between them. Sera from 127 cancer patients and 91 non-cancer patients were tested, using the reaction with heterologous anti-CEA as positive control and as a reference point. In 46 cases (21%) the sera were reactive in this system, and 43 of them were of Blood Group O. However, there was no difference between the cancer patients and the normal controls. The antigenic determinant involved in this reaction is not the Blood Group A specificity but, most probably, a polypeptide common to CEA and A (as shown in the following publication). In addition, trials for the elimination of the non-tumour-specific reaction, by absorption or inhibition, failed to disclose a tumour-specific one. The value of the ADCC assay in monitoring human tumour immunity, and possible ways of eliminating reactivity to normal antigens in this system, are discussed in the light of these findings.


*Br. J. Cancer* (1979) **40**, 234

### ANTIBODY-DEPENDENT CELLULAR CYTOTOXICITY TO HUMAN COLON-TUMOUR CELLS. I. LACK OF TUMOUR SPECIFICITY IN A POPULATION STUDY

J. SHOHAM AND M. COHEN

*From The Genetic Institute, Sheba Medical Centre, Tel-Hashomer, Israel*

Received 30 November 1978 Accepted 12 April 1979

**Summary.—The humoral and cellular components of the antibody-dependent cellular cytotoxicity (ADCC) against allogeneic human colonic tumour cell lines were evaluated. The 2 colon cell lines used in this study (HT-29 and ACC-20) were found by immunofluorescence to have carcinoembryonic antigen (CEA) on their surface, and to become sensitive to the lytic effect of unstimulated lymphocytes after coating with heterologous anti-CEA. This reaction was used to evaluate the ADCC activity of mononuclear cells from the peripheral blood of patients with gastrointestinal cancer (mostly local extensive colo–rectal). Remarkable variability was found in the lytic capability (2–50% specific lysis) of both cancer and non-cancer mononuclear cells, with no significant difference between them. Sera from 127 cancer patients and 91 non-cancer patients were tested, using the reaction with heterologous anti-CEA as positive control and as a reference point. In 46 cases (21%) the sera were reactive in this system, and 43 of them were of Blood Group O. However, there was no difference between the cancer patients and the normal controls. The antigenic determinant involved in this reaction is not the Blood Group A specificity but, most probably, a polypeptide common to CEA and A (as shown in the following publication). In addition, trials for the elimination of the non-tumour-specific reaction, by absorption or inhibition, failed to disclose a tumour-specific one. The value of the ADCC assay in monitoring human tumour immunity, and possible ways of eliminating reactivity to normal antigens in this system, are discussed in the light of these findings.**

ANTIBODY-DEPENDENT cellular cytotoxicity (ADCC) is a well defined *in vitro* reaction (Lovchik & Hong, 1977; Perlman, 1976) of unknown biological significance. The specificity of the reaction is determined by the antibodies that coat the target cells. The nonspecific cellular effector component is the K lymphocyte (Perlman, 1976) or sometimes the macrophage (Kohl *et al.*, 1977) or polymorphonuclear leucocyte (Gale & Zighelboim, 1975). All of them recognize the Fc portion of the coating antibody.

This reaction has been implicated in various *in vivo* immune processes, including those against tumours (Lamon *et al.*, 1976), virus-infected cells (Pearson & Orr, 1976), transplants (Jeannet & Vassalli, 1976) and autoimmunity (Feldman *et al.*, 1976). However, although there is some evidence that ADCC does occur in these conditions, it has been impossible so far to demonstrate directly their relative importance among the multitude of immune reactions taking place *in vivo*. As far as tumour immunity is concerned, serum and lymphocyte cooperation was demonstrated *in vitro* in mice bearing Moloney sarcoma virus-induced or 3-methylcholanthrene-induced sarcomas and mammary-tumour virus-induced adenocarcinoma (Pollack *et al.*, 1972), as well as in rats with Gross virus-induced lymphoma (Ortiz de Landazuri *et al.*, 1974). Successful inhibition of murine neuroblastoma by ADCC was demonstrated in a modified Winn's test

Correspondence to: Dr J. Shoham, The Genetic Institute, Sheba Medical Centre, Tel-Hashomer, Israel.

(Byfield *et al.*, 1976). With human tumours the condition is much less clear, and there are few publications suggesting that specific ADCC might occur in a limited number of cases (Kodera & Bean, 1975; Hellström *et al.*, 1973; Hersey *et al.*, 1973; Hakala & Lange, 1974). However, the remarkable sensitivity of this reaction to low antibody levels (Zighelboim *et al.*, 1973; Perlman, 1976) justify further evaluation of its applicability to the monitoring of antitumour activity in humans.

The present study was undertaken in order to use the ADCC reaction in the human colonic-tumour system using serum or lymphocytes from patients and matched controls against human colonic tumour cell lines. Our observations, and those of others (Von Kleist *et al.*, 1975), suggest that carcinoembryonic antigen (CEA) and other surface antigens are present unchanged on these cells, despite their long period *in vitro*. In addition, their morphology in culture and histology as tumours in nude mice suggest that their tumour characteristics are well preserved. Preliminary distinct ADCC reactions with both heterologous anti-CEA and some patients' sera encouraged further study in this direction.

### MATERIALS AND METHODS

*Cells and cell cultures.*—The cell lines used in these experiments originated from adenocarcinoma of the colon (HT-29, ACC-20), malignant melanoma (SK-mel, $IgR_3$, 8342, 8322) and adenocarcinoma of the endometrium (Endo 1-11 and Endo-2). ACC-20 is a cell line developed in our laboratory from malignant effusion of a patient with cancer of the colon. The other cell lines were kindly given to us by Dr Brunner (SK-mel and $IgR_3$) and Dr Sordat (HT-29 and the endometrial lines), both of the Swiss Institute for Experimental Cancer Research, Lausanne, and Dr G. Moore (8342, 8322), Denver General Hospital, Denver, Colorado, U.S. Only colonic-tumour cells were positive for cell-surface immunofluorescence with anti-CEA serum. Cells were cultured in plastic flasks (Falcon) in Dulbecco's modified Eagle's medium, high glucose (GIBCO) with 10% foetal calf serum (GIBCO). Routine passage of cells in a monolayer (HT-29, ACC-20, $IgR_3$, 8342, 8322) was made once a week with 0·25% trypsin solution (1:300) or by dilution for cells in suspension (SK-mel and endometrial lines). There was no detectable bacterial, fungal or mycoplasmal contamination of cultures used in these studies.

*Sera.*—Goat anti-CEA serum, lyophilized globulin fraction (ammonium sulphate precipitation) was kindly given to us by Dr Gold from McGill University, Montreal, Canada. This antiserum was dialysed and absorbed on a mixture of normal human lung, liver and colonic tissues and normal human serum. Normal goat serum (NGS) was used as a control. 218 samples of human sera were collected. Of those, 127 were from cancer patients (61 with local extensive colo-rectal cancer, 15 with gastric and 12 with pancreatic cancers, 23 with melanoma Stage I or II and 16 with other tumours). 91 sera were from people without cancer (38 of them healthy young donors and 53 controls matched for age, sex and also, if possible, disease states such as atherosclerotic cardiovascular disease, hypertension or diabetes mellitus).

*Mononuclear cells.*—Blood samples were drawn into heparinized syringes and allowed to settle for 30–45 min. The leucocyte-rich plasma was separated and layered on Ficoll–Hypaque solution for density separation of lymphocytes (Thorsby & Bratlie, 1970). Cells at interface were aspirated and washed ×3 in serum-free medium. These preparations contained 92–97% mononuclear cells, most of them (70–85%) with the morphological appearance of small lymphocytes. Such cell samples contained 54·7 ± 10·2% and 50·7 ± 11·7% E rosettes in normal adults and cancer patients respectively (Shoham *et al.*, to be published), and 5–10% B rosettes (with mouse red blood cells). The "null" cell population was not further analysed for the presence of non-lymphocyte white blood cells (Zucker-Franklin, 1974; Currie *et al.*, 1978). Nevertheless, we will refer conventionally to these mononuclear cell preparations as peripheral-blood lymphocytes (PBL) with the understanding that other white blood cells present in these preparations may contribute to the ADCC reaction (Kohl *et al.*, 1977; Gale & Zighelboim, 1975). Viability was tested by eosin-Y exclusion, 95–100% of the cells being viable.

*Assay of ADCC.*—Target cells (from the above-mentioned cell lines) were harvested and incubated with 200 $\mu$Ci $^{51}$Cr for 90 min at 37°C, washed ×2, incubated with goat anti-CEA, normal goat serum or human test serum, for 30 min at 37°C, then washed ×2. 100 $\mu$l of the target cells ($10^5$/ml) were mixed with 100 $\mu$l of mononuclear cells ($10^7$/ml) or with medium and incubated for 18 h. Then 200 $\mu$l medium was added to each tube, which was vortexed and centrifuged. 200 $\mu$l of the supernatant was transferred to another tube, and the results read in a Packard Auto-Gamma Spectrometer. The results were calculated according to the following formula:

$$\%\ \text{chromium release} = \frac{2\bar{A}}{A+\bar{A}} \times 100$$

Where A is the original tube (cell pellet + 200 $\mu$l residual supernatant) and $\bar{A}$ is the tube with the 200 $\mu$l transferred supernatant. Maximal release was determined on freeze-thawed cells and was found to be very close to the total count; % specific lysis = % experimental release − % spontaneous release.

### RESULTS

### *Basic features of the assay system*

Antibody-dependent cellular cytotoxicity to human colonic-tumour cells (HT-29) was demonstrated by incubating these cells with anti-CEA and then with PBL. The cytotoxicity was measured by $^{51}$Cr release. Table I contains crude data which reveal several features of the assay system. The maximum releasable $^{51}$Cr approaches very closely to the total counts per sample. As the latter value is available for each point in our assay, it was found more accurate to calculate % specific lysis from it rather than from the maximum release. The spontaneous release does not exceed 30% in HT-29 and the other cell lines chosen for assay.

Cellular cytotoxicity against uncoated cells (cell-mediated cytotoxicity, CMC) is low, and approaches the spontaneous release (Tables I and II). This is the case with most of the PBL samples tested, but not with all (Fig. 3). Similar results in both ADCC and CMC reactions were obtained with a second colonic tumour-cell line (ACC-20).

The ADCC reaction with anti-CEA is apparently immunologically specific (Table II). Normal goat serum (NGS) as well as 2 antisera to unrelated antigens were inactive in this system. Coating of melanoma cells and of endometrial tumour cells with anti-CEA did not change the effect exerted by lymphocytes *per se*. Table II represents

TABLE I.—*Crude data of the assay system*

| Incubation conditions* | ct/min | | | | % $^{51}$Cr release | |
|---|---|---|---|---|---|---|
| | Tube A | Tube $\bar{A}$ | Total count | Total release | | Mean ± s.d. |
| Maximal release | 1608 | 1531 | 3139 | 3062 | 97·5 | |
| | 1692 | 1549 | 3241 | 3098 | 95·6 | 96·4 ± 0·8 |
| | 1749 | 1613 | 3362 | 3226 | 96·0 | |
| Medium only† (spontaneous release) | 2523 | 360 | 2883 | 720 | 25·0 | |
| | 2480 | 422 | 2902 | 844 | 29·1 | 27·1 ± 1·7 |
| | 2537 | 403 | 2940 | 806 | 27·4 | |
| With PBL‡ (CMC) | 2948 | 537 | 3385 | 1074 | 31·7 | |
| | 2869 | 496 | 3365 | 992 | 29·5 | 31·2 ± 1·2 |
| | 2832 | 542 | 3379 | 1094 | 32·4 | |
| With anti-CEA then PBL (ADCC) | 2384 | 723 | 3107 | 1446 | 46·5 | |
| | 2277 | 714 | 2991 | 1428 | 47·7 | 46·5 ± 1·0 |
| | 2324 | 680 | 3004 | 1360 | 45·3 | |

* HT-29 cells were labelled with $^{51}$Cr and then subjected to either freeze-thawing (maximal release) or incubation with medium alone for 18 h (spontaneous release) with PBL with (ADCC) or without (CMC) preincubation with anti-CEA.

† Similar results with NGS or anti-CEA-coated cells without added lymphocytes.

‡ Similar results with or without preincubation with NGS.

TABLE II.—*Serum and cell controls for specificity**

| Target cells | Pre-incubated with | % $^{51}$Cr release ± s.d. Medium§ | PBL |
|---|---|---|---|
| HT-29† | anti-CEA | 27·3 ± 1·7 | 46·5 ± 0·98 |
| | NGS | 30·5 ± 1·6 | 32·7 ± 0·2 |
| | PBS | 29·2 ± 1·9 | 31·2 ± 1·2 |
| SK-mel‡ | anti-CEA | 18·8 ± 1·8 | 29·5 ± 1·1 |
| | NGS | 20·9 ± 0·4 | 31·7 ± 0·7 |
| | PBS | 18·2 ± 1·1 | 26·8 ± 1·6 |

* Conditions as in Table I.

† ACC-20 gave similar results.

‡ Two other melanoma cell lines and 2 endometrial cell lines also exhibited the same lytic effect with or without coating with anti-CEA.

§ Spontaneous release.

FIG. 1.—Titration of the ADCC activity of goat anti-CEA. $10^4$ $^{51}$Cr-labelled HT-29 cells were coated with reconstituted preparation of anti-CEA (8 mg protein/ml = log 1) on serial $\log_{10}$ dilutions of it (○——○) or with normal goat serum NGS (□----□) and then with $10^6$ lymphocytes.

the results with one of the melanoma cell lines as a control.

*Dose–effect relationships*

The lytic effect in this assay is dose-related both to the serum concentration and to the amount of lymphocytes. Using 10-fold dilutions of the anti-CEA serum (originally reconstituted to 8 mg/ml protein) with constant lymphocyte: target ratio (100:1 with $10^4$ target cells) we obtained an approximately linear decrease in the percentage lysis (Fig. 1). The lytic effect was detectable up to a 1000-fold

FIG. 2.—ADCC reaction as a function of lymphocyte/target ratio. $10^4$ HT-29 cells were incubated with 1 mg/ml anti CEA (○——○) or NGS (□----□), then with varying amounts of PBL ($10^5$–$10^6$).

dilution. In further experiments we used anti-CEA in a concentration of 1 mg/ml protein.

The lymphocyte-dose-dependency of the reaction is shown in Fig. 2. There was a sharp decline in % lysis when the number of lymphocytes was changed from $10^6$ to $5 \times 10^5$ lymphocytes. The following experiments were carried out with $10^6$ PBL.

*Patient lymphocytes as the tested variable*

PBL from 41 healthy subjects or non-cancer patients and 25 with gastrointestinal (GI) cancer were tested in this assay for both ADCC against anti-CEA-coated HT-29 cells and CMC against uncoated cells (Fig. 3). Most of the patients (21/25) had local extensive large-bowel tumour without metastasis, and did not receive chemotherapy or radiotherapy at the test time. The score in Fig. 3 reflects the fact that the ability of PBL to participate in ADCC fluctuates from the barely measurable to about 60% specific lysis. However,

FIG. 3.—ADCC and CMC activity of PBL from people without cancer and patients with gastro-intestinal (GI) cancer, mostly (21/25) local extensive colo-rectal adenocarcinoma before radiation or chemotherapy. In the non-cancer group the activity of the individual samples was compared to that of pools from 2 donors.

in most of the cases the reaction was clearly demonstrated (10% or more specific lysis in 33/41 non-cancer and 23/25 cancer patients). In contrast, with uncoated target cells there was no measurable lymphocytotoxicity in 27/41 and 16/27 of the samples respectively. Only 6/41 and 5/27 had 10% or more specific lysis. Statistical analysis by Wilcoxon's test indicates that there is no difference in both ADCC and CMC between the non-cancer and cancer group.

*Patient sera as the tested variable*

HT-29 cells were incubated with human sera taken from non-cancer or cancer patients, and then with PBL. Anti-CEA-coated HT-29 cells served as a positive control. Here also we obtained a spectrum of activities from zero (equal to spontaneous release) to those approaching maximal release. However, the variability introduced by the lymphocytes (Fig. 3) precludes comparison of results with different lymphocyte preparation.

Pooling lymphocytes (usually from 2 donors) enabled us to compare larger numbers of sera under uniform conditions without significantly changing the ADCC reactivity (Fig. 3) and this served as a partial answer to this problem. It has to be mentioned, however, that CMC of pooled PBL against uncoated HT-29 cells was significantly higher ($P < 0.01$) than that of unpooled PBL. Moreover, anti-CEA was used as a reference serum for each PBL sample, and the results with the patients' sera were related to those of anti-CEA by dividing the % specific lysis (SL) of each one of the tested sera by that of anti-CEA. The results were regarded as the "cytotoxicity index" (CI). The CI of anti-CEA was, therefore, always 1·0 (Table III).

The CI introduced a remarkable uniformity to the results of each serum tested with several PBL specimens, or even in different experiments.

Sera from 218 subjects were tested (91 healthy individuals or non-cancer patients and 127 with cancer of various origins). It was found in repeated experiments that a CI $< 0.7$ is insignificant, as compared to the background, and therefore a CI of 0·7 was taken as the boundary between positive and negative reactions. By this definition 46/218 (21%) of the sera were positive, of which 19/91 (21%) were from the non-cancer group and 26/127 (20·4%) in the cancer group. The difference is obviously insignificant.

When the results were correlated with the blood group of these subjects (Fig. 4), it emerged that almost all the reactors (43/46) were of Blood Group O. However, only 43/83 (51%) of the Blood Group O sera reacted. Blood Group A sera did not react at all, whereas 2 sera of Blood Group B and 1 of Group AB also reacted. The results with Blood Group O sera were further analysed by the Mann–Whitney *U* test as a continuous score without regarding the 0·7 index cut. This analysis also confirmed the absence of difference between cancer and non-cancer patients. Moreover, when the results of Blood-Group O cancer sera were scored separately according to the primary tumour, no difference among them could be demon-

TABLE III.—*Conversion of % specific lysis (SL) to cytotoxicity index (CI) using the response with goat anti-CEA as a reference value*

| | | Serum† | | | | | | | |
|---|---|---|---|---|---|---|---|---|---|
| | | anti-CEA | | S-8 | | S-22 | | S-78 | |
| Exp. | PBL* sample | SL | CI | SL | CI | SL | CI | SL | CI |
| 1 | A | 45·7 | 1·0 | 36·2 | 0·79 | 40·0 | 0·88 | ND | |
| | B | 8·4 | 1·0 | 7·2 | 0·86 | 7·8 | 0·93 | ND | |
| | C | 34·5 | 1·0 | 29·5 | 0·85 | 27·1 | 0·78 | 11·8 | 0·34 |
| | D | 22·1 | 1·0 | 24·6 | 1·13 | 18·3 | 0·82 | ND | |
| 2 | E | 18·9 | 1·0 | 16·3 | 0·86 | ND | | 5·1 | 0·26 |
| | F | 27·4 | 1·0 | 25·1 | 0·92 | ND | | ND | |
| 3 | G | 12·8 | 1·0 | ND | | ND | | 4·7 | 0·36 |

* Seven PBL samples were used in 3 experiments for the ADCC assay; anti-CEA was included in all the experiments and the results with it are used as reference CI (1·0). The results with the human sera are related to it. S-8 and S-22 are 2 serum samples taken from the same patient on 2 different occasions and are positive (CI > 0·7, see text) and S-78 is from another patient and negative (CI < 0·7).

FIGURE 4.—Activity of sera from cancer patients (C) and non-cancer (NC), arranged according to the blood group and expressed as cytotoxicity index.

FIG. 5.—Activity of sera from cancer patients with different tumours, and Blood Group O, expressed as cytoxicity index.

strated (Fig. 5). The single positive Group AB serum was from multiparous women, and was found to contain HLA antibodies.

The titre of the positive human sera was lower than that of anti-CEA, and approached background activity with dilutions of $\frac{1}{16}$ or $\frac{1}{32}$ (Fig. 6), without a difference between cancer and non-cancer sera, even when initially highly ADCC-active sera were selected.

### DISCUSSION

The 2 components of the ADCC (cellular and humoral) were tested for their usefulness in the monitoring of human tumour immunity in allogeneic combination with colonic tumour cell lines.

As far as the cellular aspect is concerned, there is remarkable variability in the ability of mononuclear cells of different subjects to participate in the ADCC reaction. Such variability has also been found in other studies (Lovchik & Hong, 1977; Korithavongs *et al.*, 1974). However, the cells of healthy people and GI cancer patients do not differ in this characteristic in our system, which tests mostly patients with local extensive colo-rectal cancer.

FIGURE 6.—Titration of ADCC activity of human positive sera of 3 healthy persons (▲——▲) or 3 cancer patients (●——●). The patient sera were selected for high initial cytotoxicity. The dashed horizontal lines represent the results with anti-CEA (defined as CI = 1·0) and NGS, representing background lysis.

Other patients with more advanced disease also showed the same pattern of distribution (data not shown). There is no agreement among other workers regarding this activity in cancer patients. The works of Lovchik & Hong (1977), Elhilali *et al.* (1976) and Peter *et al.* (1975*a*) support our observations, whereas Ting & Terasaki (1974) found depressed ADCC-effector activity in cancer patients. It has to be emphasized that the Ficolle–Hypaque-separated mononuclear cell preparations may contain substantial numbers of macrophages (Zucker-Franklin, 1974) and chloroacetate-esterase-positive cells and that the percentage of the last-mentioned cells may be especially high in cancer patients (Currie *et al.*, 1978). Although such cells may participate in ADCC (Kohl *et al.*, 1977; Gale & Zighelboim, 1975) along with K lymphocytes, quantitative and kinetic differences in this activity may exist among them. Taking all these data together, and in view of the remarkable variability in this activity in both healthy persons and cancer patients, we do not feel that testing for lymphocyte activity in ADCC has a place in monitoring the immune status of cancer patients.

Twenty-one per cent of the sera tested were found to be reactive in this system, and to cause colonic tumour cell lysis to about the same degree as heterologous anti-CEA does with undiluted serum. The titre of the heterologous anti-CEA was about 20-fold higher than that of the positive allogeneic sera. However, once again no difference was found between cancer patients and normal controls. Further analysis revealed that almost all the positive sera (43/46) were of Blood-Group O persons, with 2 of Group B, 1 of Group AB and none of Group A. However, not all O-type sera were reactive. Thus, the reaction was apparently against A antigen or A-like determinant on the CEA molecule, which may or may not mask a more specific reaction towards other determinants on the CEA molecule or other colonic-tumour associated antigens. These possibilities are further analysed in the following publication (Shoham & Cohen, 1979), which brings evidence that the reaction observed is to an antigenic determinant common to CEA and A, and maybe normal colon antigen too. This determinant most probably resides in the protein portion of the molecule. The inhibition of this common activity did not expose tumour-specific activity.

Lymphocyte dependent antibody (LDA) activity was looked for in other human tumour-cell systems. Hellström *et al.* (1973) showed increased lymphocyte-mediated tumour-cell destruction in their test systems with allogeneic combination of sera from 7 cancer patients (with different tumours) out of a "much larger patient material" (unspecified) with no reference to normal antigens. The same reservation applies to the work of Hakala & Lange (1974), who found LDA activity in 2/40 transitional-cell carcinoma patients. In other publications a large panel of allogeneic target-tumour cells of the same histological type was used in order to solve the problem of immune specificity. Ferrone & Pellegrino (1977) used several melanoma cell lines in a complement-dependent microcytotoxicity assay. They failed to find melanoma-specific activity as compared to serum activity in other cancer patients or correlation to disease stage. Hersey *et al.* (1973) found LDA in sera from AML patients to a panel of allogeneic AML myeloblasts, which they felt may be directed to a leukaemia-associated antigen. However, some of these patients had received immunotherapy with allogeneic cells, and all of them had been given multiple transfusions. Thus it is more plausible that the observed activity was related to HLA antigens. This notion is further supported by the recent work of Gale & MacLennan (1977). Autochthonous combinations may avoid this confusion. Kodera & Bean (1975) used such combinations and found LDA activity in 4/16 patients, which was apparently related to disease state. A similar study by Peter *et al.* (1975*b*) in a smaller group of patients failed to show such activity. However, the numbers in the last 2 studies are too small to warrant any firm conclusion on the significance of this activity, and the difficulties encountered in using autochthonous combinations preclude their large-scale use. An alternative approach is to eliminate the activity to HLA and blood-group antigens by selective absorption or by inhibition with Fab fragments of appropriate polyspecific serum. The potential of this approach is demonstrated in the following publication (Shoham & Cohen, 1979). However, in the particular case of colonic tumour cells it failed to expose any specific tumour activity.

This work was supported in part by research grants 6/74 and 3/75 from the Israel Cancer Association.

### REFERENCES

BYFIELD, J. E., ZERUBAVEL, R. & FONKALSRUD, E. E. (1976) Murine neuroblastoma cured *in vivo* by antibody dependent cellular cytotoxicity reaction. *Nature*, **264**, 783.

CURRIE, G. A., HEDLEY, D. W., NYHOLM, R. E. & TAYLOR, S. A. (1978) Contamination of mononuclear cell suspensions obtained from cancer patients by the Boyum method. *Br. J. Cancer*, **38**, 555.

ELHILALI, M. M., BRITTON, S., BROSMAN, S. & FAHEY, J. L. (1976) Critical evaluation of lymphocyte function in urological cancer patients. *Cancer Res.*, **36**, 132.

FELDMAN, J. L., BECKER, M. J., MOULSOPOULOS, H. & 4 others (1976) Antibody dependent cell mediated cytotoxicity in selected autoimmune diseases. *J. Clin. Invest.*, **58**, 173.

FERRONE, S. & PELLEGRINO, M. A. (1977) Cytotoxic antibodies to cultured melanoma cells in the sera of melanoma patients. *J. Natl Cancer Inst.*, **58**, 1201.

GALE, D. G. & MACLENNAN, I. C. M. (1977) Cytotoxic antibody in acute myeloblastic leukaemia during immunotherapy: lack of tumour specificity. *Br. J. Cancer*, **35**, 280.

GALE, R. P. & ZIGHELBOIM, J. (1975) Polymorphonuclear leukocytes in antibody dependent cellular cytotoxicity. *J. Immunol.*, **114**, 1047.

HAKALA, T. T. & LANGE, P. H. (1974) Serum induced lymphoid cell mediated cytotoxicity to human transitional cell carcinoma of the genitourinary tract. *Science*, **184**, 795.

HELLSTRÖM, I., HELLSTRÖM, K. E. & WARNER, G. A. (1973) Increase of lymphocyte mediated cell destruction by certain patient sera. *Int. J. Cancer*, **12**, 348.

HERSEY, P., MACLENNAN, I. C. M., CAMPBELL, A. C., HARRIS, R. & FREEMAN, C. B. (1973) Cytotoxicity against human leukemic cells. *Clin. Exp. Immunol.*, **14**, 159.

JEANNET, M. & VASSALLI, P. (1976) The role of lymphocyte dependent antibody in kidney transplantation. *Transplantation*, **22**, 493.

KODERA, Y. & BEAN, M. A. (1975) Antibody dependent cell-mediated cytotoxicity for human monolayer target cells bearing blood group and transplantation antigens for melanoma cells. *Int. J. Cancer*, **16**, 579.

KOHL, S., STARR, S. E., OLESKE, J. M., SHORE, S. L., ASHMAN, R. B. & NAHMIAS, A. J. (1977) Human monocyte macrophage mediated, antibody dependent cytotoxicity to herpes simplex virus infected cells. *J. Immunol.*, **118**, 729.

KORITHAVONGS, T., HOLLMAN, V. C. & DOSSETTOR, J. B. (1974) Effector cell activity in antibody-mediated cell-dependent immune lympholysis. *J. Immunol.*, **113**, 1178.

LAMON, E. W., HALE, P. & WHITTEN, H. D. (1976) Antibody dependent cell mediated cytotoxicity with autochthonous lymphocytes and sera after infection with Moloney sarcoma virus. *J. Natl Cancer Inst.*, **56**, 349.

LOVCHIK, J. C. & HONG, R. (1977) Antibody dependent cell-mediated cytolysis: analysis and projections. *Prog. Allergy*, **22**, 1.

ORTIZ DE LANDAZURI, M., KEDAR, E. & FAHEY, J. L. (1974) Synergistic cooperation between isoantiserum and immune lymphoid cells: *in vitro* studies with a synergistic rat lymphoma. *J. Immunol.*, **112**, 2102.

PEARSON, G. R. & ORR, T. W. (1976) Antibody dependent lymphocyte cytotoxicity against cells expressing Epstein–Barr virus antigens. *J. Natl Cancer Inst.*, **56**, 485.

PERLMAN, P. (1976) Cellular immunity: antibody-dependent cytotoxicity (K-cell activity). *Clin. Immunobiol.*, **3**, 107.

PETER, H. H., PARIE-FISCHER, J., FRIDMAN, W. H. & 4 others (1975*a*) Cell mediated cytotoxicity *in vitro* of human lymphocytes against a tissue culture melanoma cell line (IgR3). *J. Immunol.*, **115**, 539.

PETER, H. H., KALDEN, J. R., SEELAND, P., DIEHL, V. & ECKERT, G. (1975*b*) Humoral and cellular immune reactions *in vitro* against allogeneic and autologous human melanoma cells. *Clin. Exp. Immunol.*, **20**, 193.

POLLACK, S., HEPPNER, G., BRAUN, R. J. & NELSON, K. (1972) Specific killing of tumor cells *in vitro* in the presence of normal lymphoid cells and sera from hosts immune to the tumor antigens. *Int. J. Cancer*, **9**, 316.

SHOHAM, J. & COHEN, M. (1979) Antibody-dependent lymphocyte cytotoxicity to human colon-tumour cells. II. Analysis of the antigens involved. *Br. J. Cancer*, **40**, 244.

THORSBY, E. & BRATLIE, A. (1970) A rapid method for preparation of pure lymphocyte suspensions. In *Histocompatibility Tcsting*. Ed. P. Terasaki. Copenhagen: Munksgaard. p. 655.

TING, A. & TERASAKI, P. I. (1974) Depressed lymphocyte mediated killing of sensitized targets in cancer patients. *Cancer Res.*, **34**, 2694.

VON KLEIST, S., CHANY, E., BURTIN, P., KING, M. & FOGH, J. (1975) Immunohistology of antigeneic patterns of a continuous cell line from a human colon tumour. *J. Natl Cancer Inst.*, **55**, 555.

ZIGHELBOIM, J., BONAVIDA, B. & FAHEY, J. L. (1973) Evidence for several cell populations active in antibody dependent cellular cytotoxicity. *J. Immunol.*, **111**, 1737.

ZUCKER-FRANKLIN, D. (1974) The percentage of monocytes among "mononuclear" cell fractions obtained from normal human blood. *J. Immunol.*, **112**, 234.